# Supplementary material for: Palmitoylation prevents B7-H4 lysosomal degradation sustaining tumor immune evasion
Source: Nat Commun. 2025 May 8;16:4254. doi: 10.1038/s41467-025-58552-5 (PMC12062253; doi:10.1038/s41467-025-58552-5)
Supplement: Supplementary file 1 — Supplementary Information [file 41467_2025_58552_MOESM1_ESM.pdf]

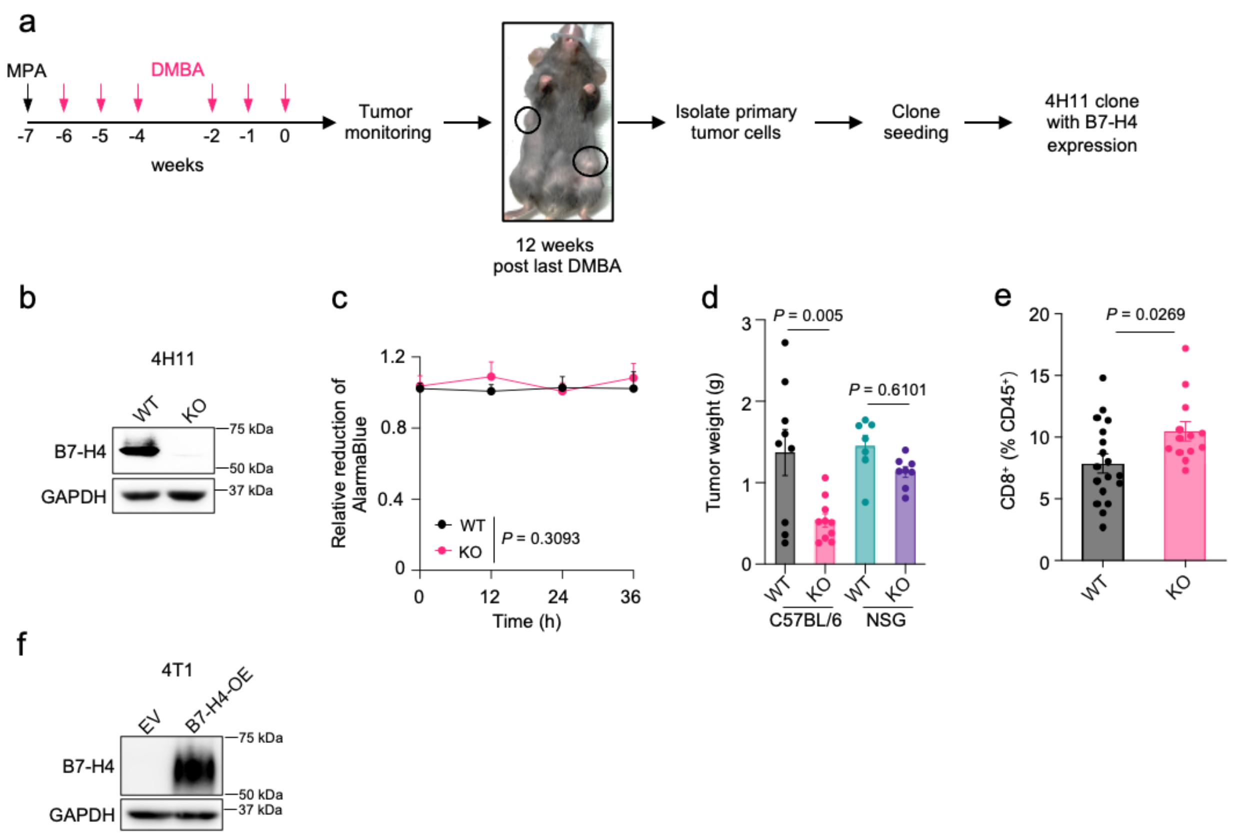

Supplementary Fig. 1: B7-H4 in breast cancer cells promotes tumor progression.

a Schematic diagram shows the generation of a 4H11 clone that expresses endogenous B7-H4 from the MPA-DMBA tumor model. MPA, medroxyprogesterone acetate. DMBA, 7, 12-dimethylbenz[a]anthrancene.

b Immunoblot for B7-H4 in 4H11 control (WT) and B7-H4 knockout (KO) cells.

c In vitro cell proliferation assay for WT and KO 4H11 cells from three independent tests. Data are shown as mean ± SEM. Statistical analysis was performed using two-way ANOVA.

d Tumor weights of B7-H4 KO (n = 10 in C57BL/6 mice and n = 8 in NSG mice) and WT (n = 9 in C57BL/6 mice and n = 7 in NSG mice) 4H11 tumors in immune-competent and NSG mice. Pooled data from 2 independent experiments. Subcutaneous implantation was conducted in 10-week-old female C57BL/6 or NSG mice. Data are shown as mean ± SEM. Statistical analysis was performed using one-way ANOVA with Tukey's multiple comparison test.

e Flow cytometry analysis of tumor-infiltrating $CD8^+$ T cells in subcutaneous 4H11 WT (n = 18) and B7-H4-KO (n =13) tumor-bearing mice.4H11 tumor-bearing mice. Pooled data from 2 independent experiments. Subcutaneous implantation was conducted in 10-week-old female C57BL/6 mice. Data are shown as mean ± SEM. Statistical analysis was performed using two-tailed Student's t test.

f Immunoblot for B7-H4 overexpression in 4T1 cells.

Source data are provided as Source Data File.

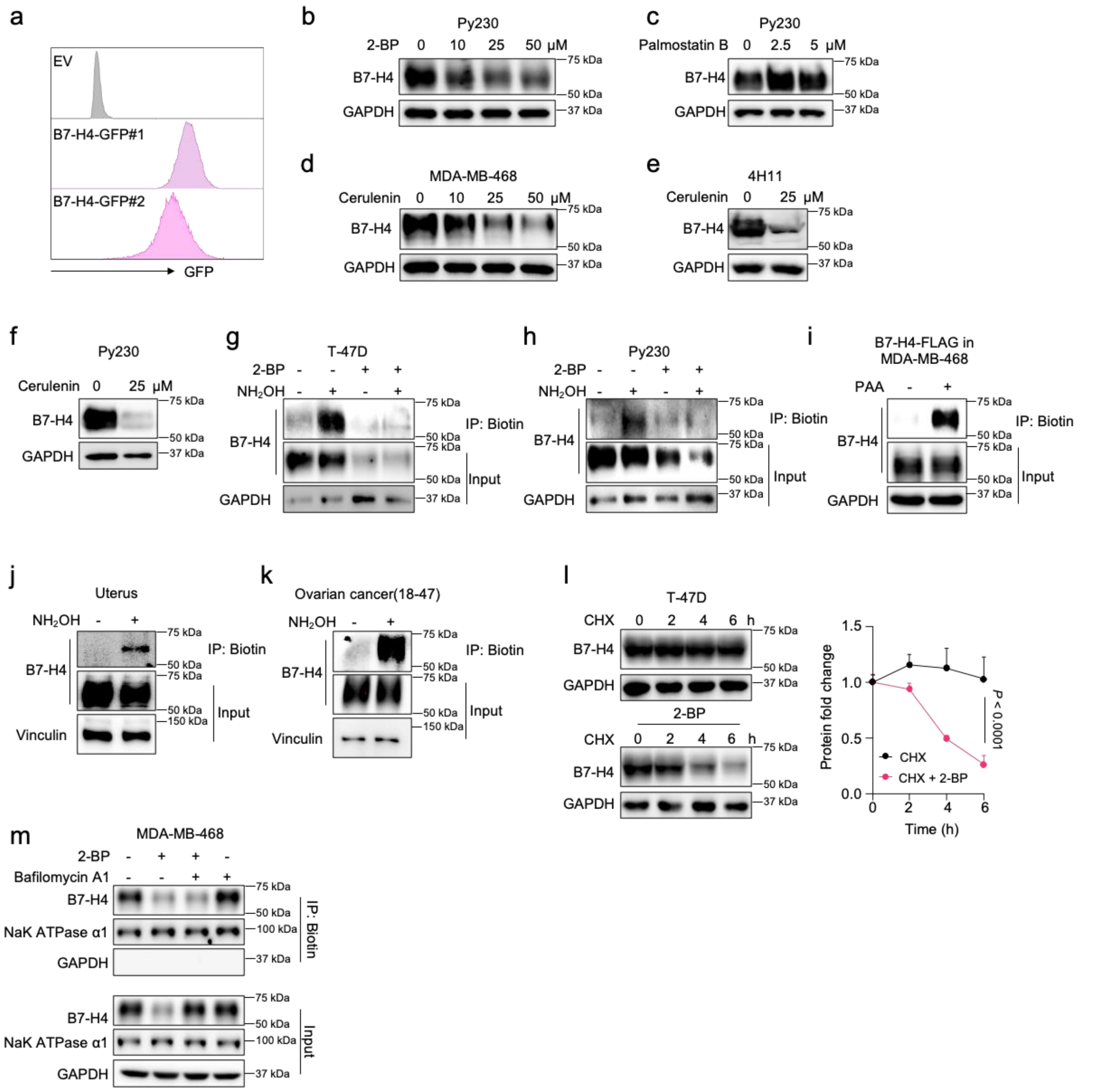

Supplementary Fig. 2: B7-H4 palmitoylation prevents its lysosomal degradation.

a Representative image of flow cytometry detection of two B7-H4-GFP expressing clones isolated from MDA-MB-468 cells.

b,**c** Immunoblot for B7-H4 in Py230 cells treated with 2-BP (**b**) or palmostatin B (**c**) at the indicated concentrations for 24 hours.

d-f Immunoblot for B7-H4 in MDA-MB-468 (**d**), 4H11 (**e**), and Py230 (**f**) cells treated with cerulenin at the indicated concentrations for 24 hours.

g,**h** Endogenous B7-H4 palmitoylation in T-47D (**g**) and Py230 (**h**) cells treated with or without (w/o) 2-BP (100 μM) determined by ABE assay.

i Palmitoylation of B7-H4 overexpressed in MDA-MB-468 cells determined by Click-iT assay. PAA, 15-azidopentadecanoic acid.

j,**k** Biotin-acyl-exchange (ABE) detects endogenous B7-H4 palmitoylation in normal mouse uterine (**j**) and human ovarian cancer (**k**) tissues.

l Representative immunoblot for B7-H4 and protein level quantification from three independent tests in T-47D cells examined by cycloheximide (CHX) chase assay w/o 2-BP (100 μM). Data are shown as mean ± SEM. Statistical analyses were performed using two-way ANOVA with Sidak's multiple comparisons test.

m Immunoblot for cell surface B7-H4 in MDA-MB-468 cells treated with 2-BP (50 μM) and/or bafilomycin A1 (100 nM) for 24 hours.

Source data are provided as Source Data File.

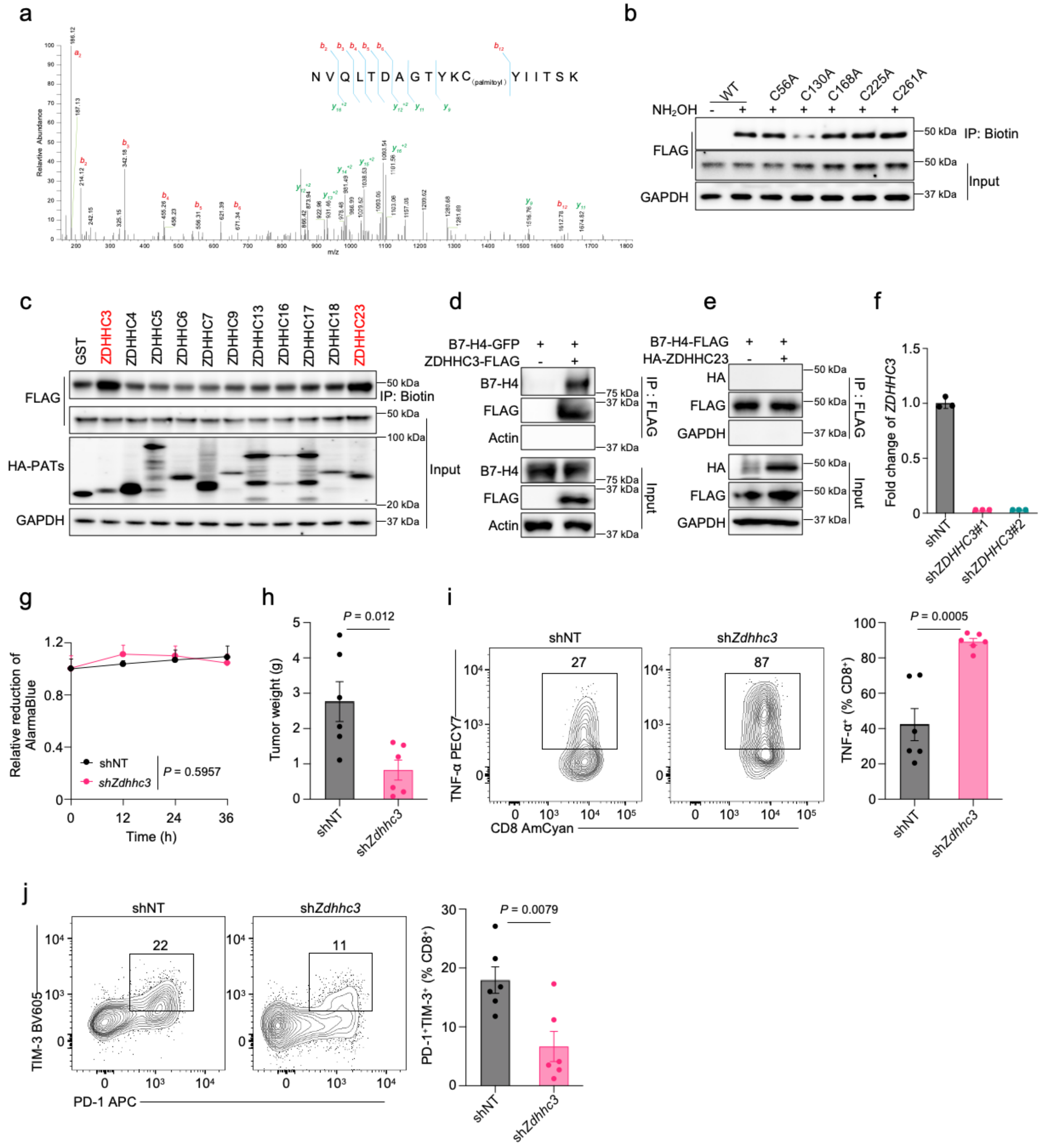

Supplementary Fig. 3: ZDHHC3 catalyzes B7-H4 palmitoylation at Cys130 impairing tumor immunity.

a Mass spectrometry identifies B7-H4 palmitoylation at Cys130.

b Palmitoylation of B7-H4 mutants in 293T cells detected by ABE assay.

c Representative immunoblot for screening palmitoyltransferase of B7-H4 by ABE assay.

d Interaction between B7-H4-GFP and ZDHHC3-Flag in 293T cells.

e Co-immunoprecipitation to detect the possible interaction between B7-H4 and ZDHHC23 in 293T cells.

f mRNA levels of ZDHHC3 in T-47D ZDHHC3 knockdown cells. Data are shown as mean ± SD.

g In vitro cell proliferation of control (shNT, non-targeting shRNA) and Zdhhc3 knockdown 4H11 cells from three independent tests. Data are shown as mean ± SEM. Statistical analysis was performed using two-way ANOVA with Sidak's multiple comparisons test.

h Zdhhc3 knockdown (sh*Zdhhc3*) and control (shNT) 4H11 subcutaneous tumor weights. n = 6 per group. Subcutaneous implantation was conducted in 8-week-old female C57BL/6 mice. Data are shown as mean ± SEM. Statistical analysis was performed using two-way ANOVA with Sidak's multiple comparisons test.

i,**j** Flow cytometry analysis of tumor-infiltrating TNF-α^+ (**i**) and PD-1^+TIM-3^+ (**j**) $CD8^+$ T cells in subcutaneous 4H11 tumor-bearing mice. n = 6 per group. Subcutaneous implantation was conducted in 8-week-old female C57BL/6 mice. Data are shown as mean ± SEM. Statistical analyses were performed using two-tailed Student's t test.

Source data are provided as Source Data File.

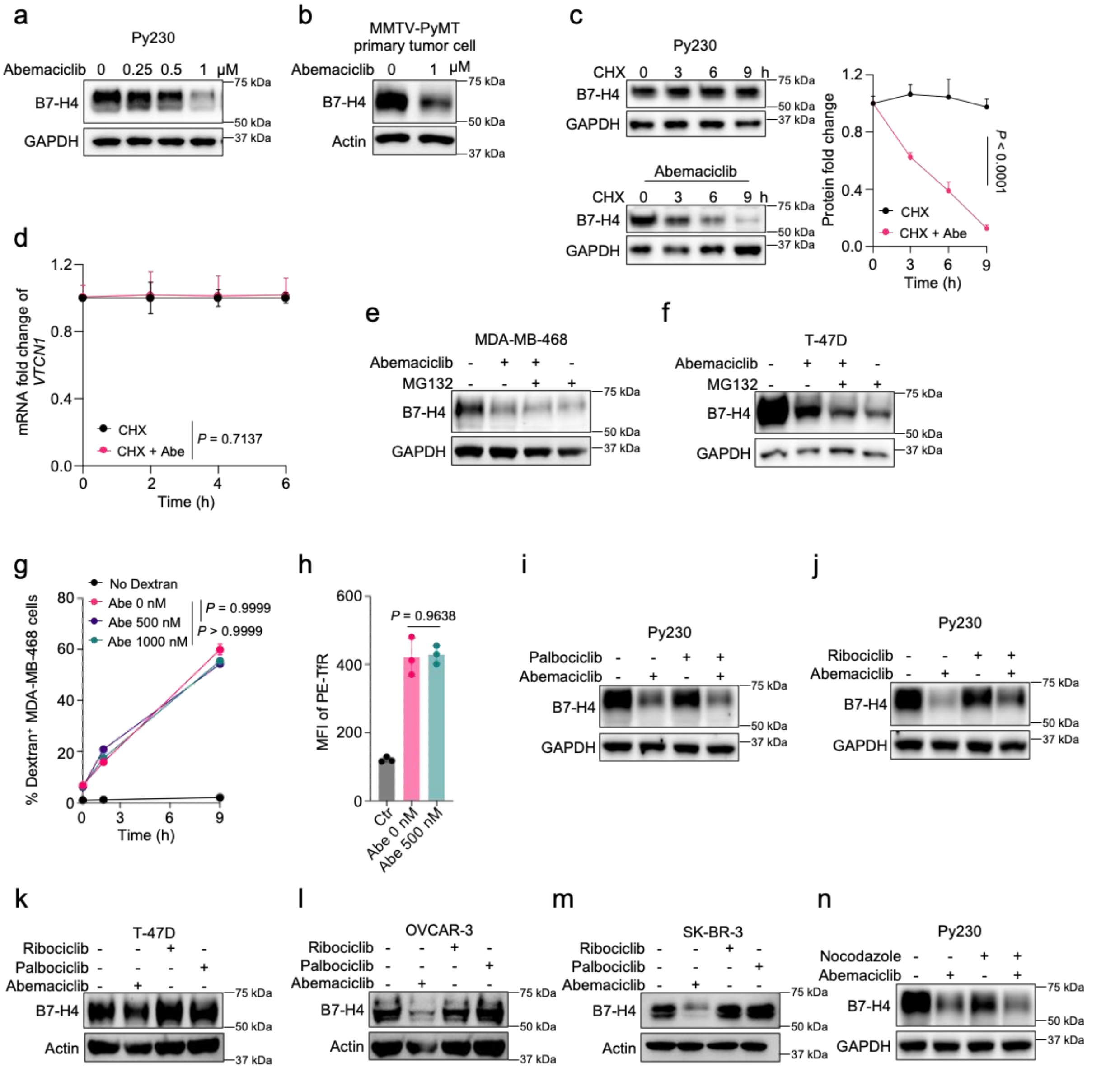

Supplementary Fig. 4: Abemaciclib promotes B7-H4 lysosomal degradation.

a,**b** Immunoblot for B7-H4 in Py230 (**a**) and MMTV-PyMT (**b**) breast cancer cells treated with abemaciclib at the indicated concentrations for 24 hours.

c Representative immunoblot for B7-H4 and protein level quantification from three independent tests in Py230 cells examined by cycloheximide (CHX) chase assay combined w/o abemaciclib (1 μM) treatment. Data are shown as mean ± SEM. Statistical analysis was performed using two-way ANOVA with Sidak's multiple comparisons test.

d mRNA fold change of B7-H4 in MDA-MB-468 cells in the setup of Fig. 4g. Data are shown as mean ± SD. Statistical analysis was performed using two-way ANOVA with Sidak's multiple comparisons test.

e,**f** Immunoblot for B7-H4 in MDA-MB-468 (**e**) and T-47D (**f**) cells treated with abemaciclib (1 μM) and/or MG132 (100 nM) for 24 hours.

g,**h** The uptake of FITC-Dextran (100 μg/mL) and the mean fluorescence intensity (MFI) of the surface transferrin receptor (TfR) in MDA-MB-468 cells treated with or without abemaciclib at the indicated concentrations for 20 hours. n = 3 per group. Data are presented as mean ± SD. Statistical analyses were conducted using two-way ANOVA (**g**) and one-way ANOVA with Dunnett's multiple comparisons test (**h**).

i,**j** Immunoblot for B7-H4 in Py230 cells treated with abemeciclib (1 μM) and/or palbociclib (1 μM) (**i**) and ribociclib (1 μM) (**j**) for 24 hours.

k-**m** Immunoblot for B7-H4 in SK-BR-3 (**k**), T-47D (**l**), and OVCAR-3 (**m**) cells treated with abemeciclib (1 μM), palbociclib (1 μM), or ribociclib (1 μM) respectively.

n Immunoblot for B7-H4 in Py230 cells treated with abemeciclib (1 μM) and/or nocodazole (1 μM) for 24 hours.

Source data are provided as Source Data File.

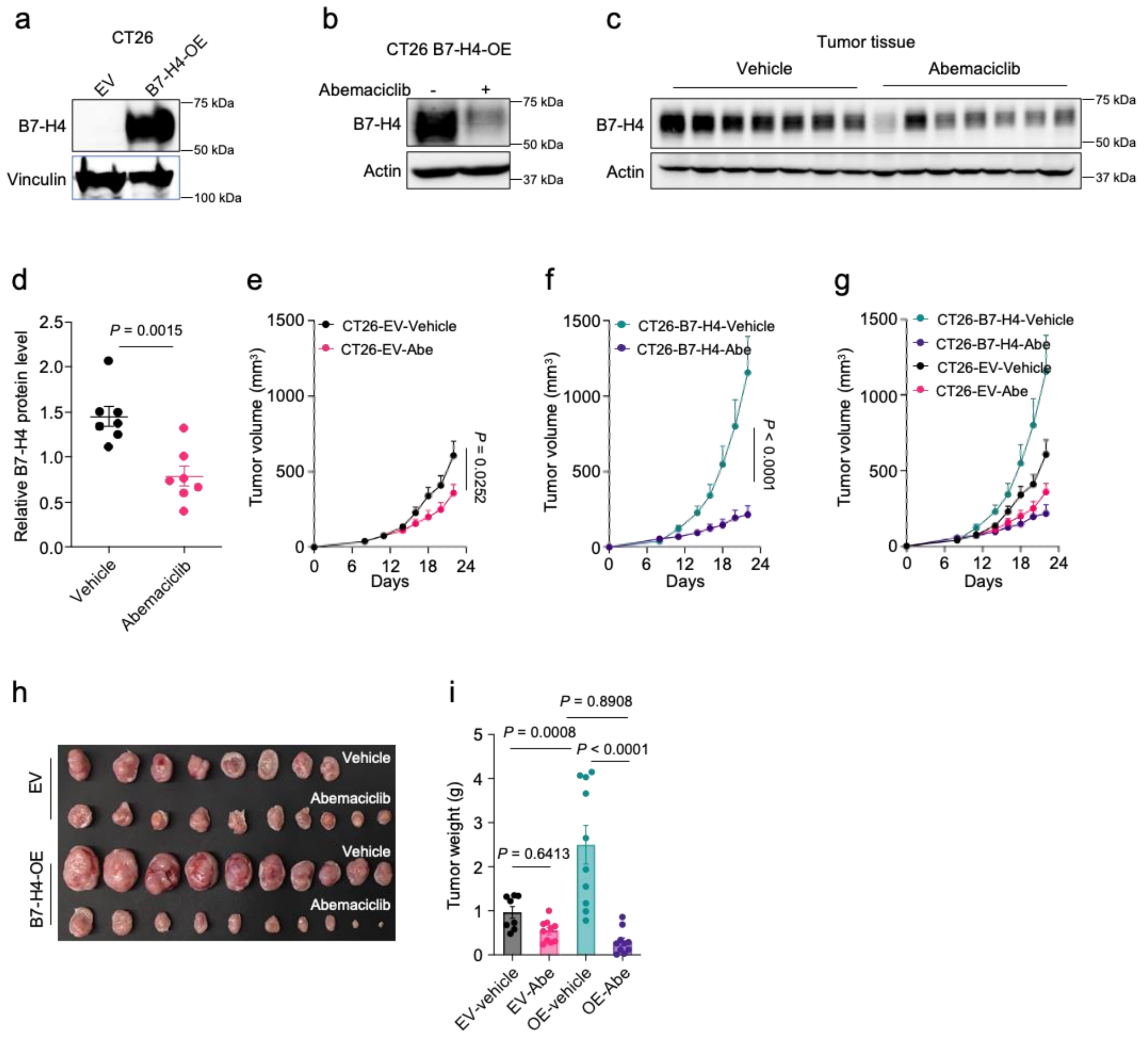

Supplementary Fig. 5: Abemaciclib enhances B7-H4 lysosomal degradation promoting tumor immunity.

a,**b** Immunoblot for B7-H4 in B7-H4 overexpressing (B7-H4-OE) CT26 cells (**a**) and in B7-H4-OE CT26 cells treated with abemaciclib (1 μM) for 24 hours (**b**).

c,**d** Immunoblot (**c**) and quantification (**d**) for B7-H4 in primary tumor cells isolated from CT-26-B7-H4 tumor-bearing mice treated with vehicle or abemaciclib (75 mg/kg, every two days). n = 7 per group. Subcutaneous implantation was conducted in 8-week-old female C57BL/6 mice. Data are shown as mean ± SEM (**d**). Statistical analysis was conducted using two-tailed Student's t test.

e-**g** CT26-EV (n= 8 for vehicle and n =10 for abemaciclib group) (**e**), or CT26-B7-H4 (n = 10 for vehicle and abemaciclib) (**f**) subcutaneous tumor growth in mice treated with vehicle or abemaciclib (75 mg/kg, every two days), and the combined growth curves (**g**).

Subcutaneous implantation was conducted in 8-week-old female C57BL/6 mice. Data are shown as mean ± SEM. Statistical analyses were conducted using two-way ANOVA (**e,f**).

h,**i** Representative image (**h**) and tumor weights (**i**) of B7-H4-OE (n = 10 for vehicle and abemaciclib) and empty vector (EV) (n = 8 for vehicle and n =10 for abemaciclib group) harboring CT26 subcutaneous tumors in mice treated with vehicle or abemaciclib (75 mg/kg, every two days). Subcutaneous implantation was conducted in 8-week-old female C57BL/6 mice. Data are shown as mean ± SEM. Statistical analyses were conducted using one-way ANOVA with Tukey's multiple comparison test.

Source data are provided as Source Data File.

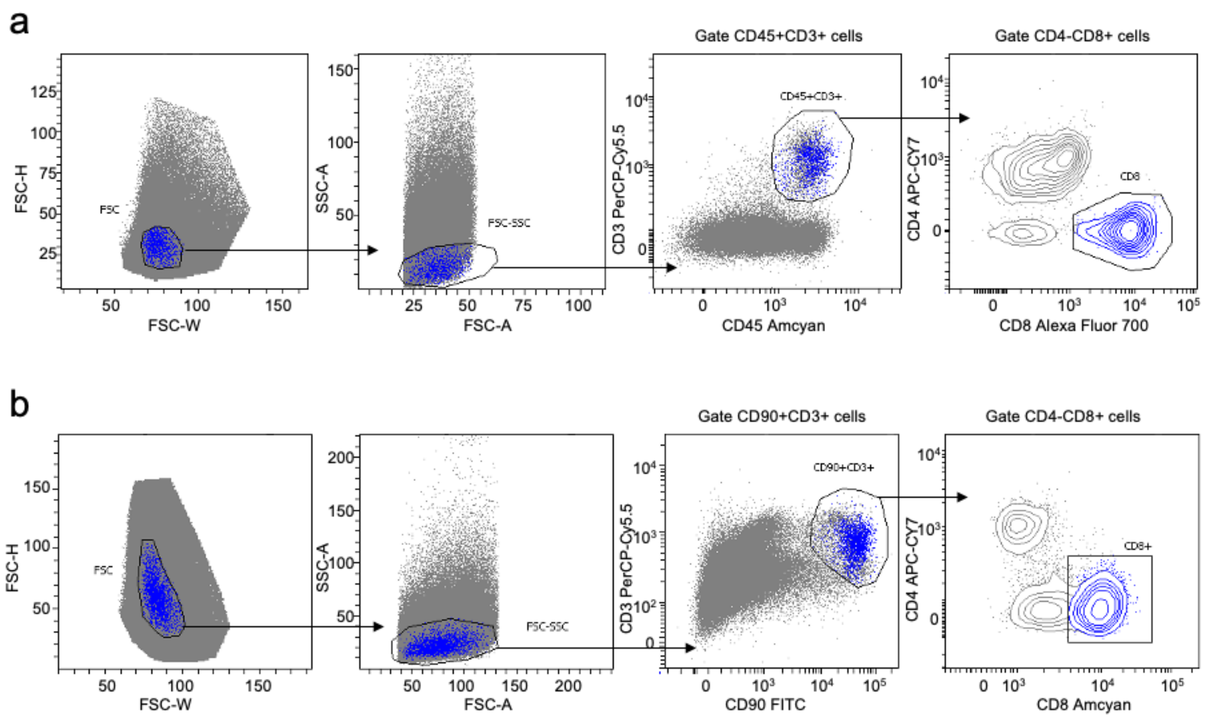

Supplementary Fig. 6: Gating strategies for flow cytometry of tumor-infiltrating CD8⁺ T cells.

a Gating strategy used to gate $CD8^+$ T cells from single-cell suspensions of the indicated tumor tissues. This strategy was applied for cell surface staining with cell-specific markers in Fig. 1d, e, k, o, and Supplementary Fig. 3j.

b Gating strategy used to gate $CD8^+$ T cells for measuring intracellular cytokine staining in Fig. 1c, 3n, and Supplementary Fig. 3i.

FSC-A: Forward scatter area; FSC-H: Forward scatter height; FSC-W: Forward scatter width; SSC-A: Side scatter area; PerCP-Cy5.5: Peridinin-Chlorophyll-protein-Cyanine5.5; FITC: Fluorescein isothiocyanate.
