# Supplementary material for: Palmitoylation prevents B7-H4 lysosomal degradation sustaining tumor immune evasion
Source: Nat Commun. 2025 May 8;16:4254. doi: 10.1038/s41467-025-58552-5 (PMC12062253; doi:10.1038/s41467-025-58552-5)
Supplement: Supplementary file 2 — Description of Additional Supplementary Files [file 41467_2025_58552_MOESM2_ESM.pdf]

Description of Additional Supplementary Files

Supplementary Data 1

Primers used the detect the mRNA levels of palmitoyltransferase.
