# Supplementary material for: Palmitoylation prevents B7-H4 lysosomal degradation sustaining tumor immune evasion
Source: Nat Commun. 2025 May 8;16:4254. doi: 10.1038/s41467-025-58552-5 (PMC12062253; doi:10.1038/s41467-025-58552-5)
Supplement: Supplementary file 4 — Reporting Summary [file 41467_2025_58552_MOESM4_ESM.pdf]

nature portfolio

Corresponding author(s): Weiping Zou

Last updated by author(s): Feb 18, 2025

Reporting Summary

Nature Portfolio wishes to improve the reproducibility of the work that we publish. This form provides structure for consistency and transparency in reporting. For further information on Nature Portfolio policies, see our Editorial Policies and the Editorial Policy Checklist.

Statistics

For all statistical analyses, confirm that the following items are present in the figure legend, table legend, main text, or Methods section.

n/a	Confirmed	
☐	☒	The exact sample size (n) for each experimental group/condition, given as a discrete number and unit of measurement
☐	☒	A statement on whether measurements were taken from distinct samples or whether the same sample was measured repeatedly
☐	☒	The statistical test(s) used AND whether they are one- or two-sided<br>*Only common tests should be described solely by name; describe more complex techniques in the Methods section.*
☐	☒	A description of all covariates tested
☐	☒	A description of any assumptions or corrections, such as tests of normality and adjustment for multiple comparisons
☐	☒	A full description of the statistical parameters including central tendency (e.g. means) or other basic estimates (e.g. regression coefficient) AND variation (e.g. standard deviation) or associated estimates of uncertainty (e.g. confidence intervals)
☐	☒	For null hypothesis testing, the test statistic (e.g. F, t, r) with confidence intervals, effect sizes, degrees of freedom and P value noted<br>*Give P values as exact values whenever suitable.*
☒	☐	For Bayesian analysis, information on the choice of priors and Markov chain Monte Carlo settings
☒	☐	For hierarchical and complex designs, identification of the appropriate level for tests and full reporting of outcomes
☒	☐	Estimates of effect sizes (e.g. Cohen's d, Pearson's r), indicating how they were calculated

Our web collection on statistics for biologists contains articles on many of the points above.

Software and code

Policy information about availability of computer code

Data collection	LSRFortessa flow cytometer was used to run samples and data was acquired and analyzed by BD FACS Diva software and Flow Jo.<br>Mass spectrometry data was collected by Q Exactive HF mass spectrometer.
Data analysis	GraphPad Prism were used for data analysis. Cufflinks/CuffDiff (version 2.2.1) was used for expression quantification, normalization, and differential expression analysis. Locally developed scripts were used to format and annotate the differential expression data output from CuffDiff. The heatmap was generated using the CummeRbund R package. Mass spectrometry data was analyzed by Proteome Discover.

For manuscripts utilizing custom algorithms or software that are central to the research but not yet described in published literature, software must be made available to editors and reviewers. We strongly encourage code deposition in a community repository (e.g. GitHub). See the Nature Portfolio guidelines for submitting code & software for further information.

Data

Policy information about availability of data

All manuscripts must include a data availability statement. This statement should provide the following information, where applicable:

- Accession codes, unique identifiers, or web links for publicly available datasets
- A description of any restrictions on data availability
- For clinical datasets or third party data, please ensure that the statement adheres to our policy

The transcriptomic data from this study is available at the Gene Expression Omnibus (GEO) under accession numbers GSE272492. The comprehensive mass spectrometry results have been submitted to the ProteomeXchange Consortium, identified by PXD054393.

Research involving human participants, their data, or biological material

Policy information about studies with human participants or human data. See also policy information about sex, gender (identity/presentation), and sexual orientation and race, ethnicity and racism.

Reporting on sex and gender	Ovarian cancer is limited to women therefore only females' material was used.
Reporting on race, ethnicity, or other socially relevant groupings	The research material was collected without selection in terms of race or ethnic origin.
Population characteristics	As the patients were not subject to any selection, they randomly represent the American population.
Recruitment	Patients with high grade serous ovarian cancer were recruited. No other selection criteria were involved.
Ethics oversight	The study was approved by the Institutional Ethics Committee of the University of Michigan, under the IRB#HUM00195340.

Note that full information on the approval of the study protocol must also be provided in the manuscript.

Field-specific reporting

Please select the one below that is the best fit for your research. If you are not sure, read the appropriate sections before making your selection.

☒ Life sciences ☐ Behavioural & social sciences ☐ Ecological, evolutionary & environmental sciences

For a reference copy of the document with all sections, see nature.com/documents/nr-reporting-summary-flat.pdf

Life sciences study design

All studies must disclose on these points even when the disclosure is negative.

Sample size	No sample size calculation was done either for in vivo or in vitro studies. For in vivo studies, n = 5-10 mice per group is sufficient to detect meaningful biological differences with good reproducibility. For in vitro studies, all the experiments were replicated at least for 3 individual, independent experiments.
Data exclusions	No data were excluded from the manuscript.
Replication	As reported in the figure legends, the findings were reliably reproduced.
Randomization	For in vivo experiments, animals were randomized based on tumor burden before they were assigned into different treatment groups, to make sure the starting tumor burden in different treatment groups was similar before treatment. All groups were age and sex matched.
Blinding	The experiments were not performed in a blinded manner as the investigator needed to know the treatment groups in order to complete the study. All data were acquired and analyzed by software with objective standard, thus blinding was not relevant to the study.

Reporting for specific materials, systems and methods

We require information from authors about some types of materials, experimental systems and methods used in many studies. Here, indicate whether each material, system or method listed is relevant to your study. If you are not sure if a list item applies to your research, read the appropriate section before selecting a response.

Materials & experimental systems

n/a	Involved in the study
☐	☒ Antibodies
☐	☒ Eukaryotic cell lines
☒	☐ Palaeontology and archaeology
☐	☒ Animals and other organisms
☒	☐ Clinical data
☒	☐ Dual use research of concern
☒	☐ Plants

Methods

n/a	Involved in the study
☒	☐ ChIP-seq
☐	☒ Flow cytometry
☒	☐ MRI-based neuroimaging

Antibodies

Antibodies used	Mouse CD45 (30-F11, BD Biosciences), CD90 (53-2.1, BD Biosciences), and CD8 (53-6.7, BD Biosciences), mouse IFN-r (Clone XMG1.2, BD Biosciences), TNF-α (Clone MP6-XT22, BD Biosciences), TCF-1 (Clone S33-966, BD Biosciences), TOX (Clone REA473, Miltenyi). Rabbit anti-B7-H4 (Abcam, ab209242), Rabbit anti-Actin (Cell Signaling Technology, 4967), Mouse anti-Flag (Genescript, A01429-100), Rabbit anti-Tubulin (Cell Signaling Technology, 2144), Mouse anti-GAPDH (Santa Cruz, sc-32233), Rabbit anti-Vinculin (Cell Signaling Technology, 4650) and Rabbit anti-Na, K-APTase a1 (Cell Signaling Technology, 3010).
Validation	All antibodies were well-recognized clones in the field and validated by the manufacturers. These antibodies are further validated and routinely used in our lab.

Eukaryotic cell lines

Policy information about cell lines and Sex and Gender in Research

Cell line source(s)	Human breast cancer cell lines MDA-MB-468 (HTB-132), SK-BR-3 (HTB-30) and T-47D (HTB-133), human ovarian cancer cell line OVCAR3 (htb-161), mouse breast cancer cell line 4T1 (CRL-2539), MCF7 (HTB-22) and Py230 (CRL-3279), mouse colon cancer cell line CT26 (CRL-2638), and human embryonic cell line HEK293T (CRL-3216) were purchased from the American Type Culture Collection (ATCC). The 4H11 cells were isolated and established from spontaneous breast tumors induced by MPA plus DMBA in C57BL/6 mice. The PyMT primary tumor cells were isolated from the mouse mammary tumor virus-polyoma middle tumor-antigen (MMTV-PyMT) tumors.
Authentication	Cell lines were not authenticated.
Mycoplasma contamination	All cell lines in our laboratory are routinely tested for mycoplasma contamination and cells used in this study are negative for mycoplasma.
Commonly misidentified lines (See ICLAC register)	No cell line used in the paper is listed in ICLAC database.

Animals and other research organisms

Policy information about studies involving animals; ARRIVE guidelines recommended for reporting animal research, and Sex and Gender in Research

Laboratory animals	The study involved the following mouse strains: NOD.SCID c-deficient (NSG) mice, wild-type C57BL/6 mice, wild-type BALB/c mice, and FVB/NJ (aged 6-10 weeks) (The Jackson Laboratory). All mice were housed under pathogen-free conditions.
Wild animals	The study did not involve wild animals.
Reporting on sex	Female mice were used in this study for the in vivo tumor experiments. As previously stated, B7-H4 is highly expressed in breast cancers. The focus of the study is on emphasizing and assessing the role of B7-H4 in breast cancer development. Consequently, female mice were selected for this study.
Field-collected samples	The study did not involve samples collected from field.
Ethics oversight	Animal studies were conducted under the approval of the University of Michigan Committee on Use and Care of Animals (PRO00011876).

Note that full information on the approval of the study protocol must also be provided in the manuscript.

Plants

Seed stocks	*Report on the source of all seed stocks or other plant material used. If applicable, state the seed stock centre and catalogue number. If plant specimens were collected from the field, describe the collection location, date and sampling procedures.*
Novel plant genotypes	*Describe the methods by which all novel plant genotypes were produced. This includes those generated by transgenic approaches, gene editing, chemical/radiation-based mutagenesis and hybridization. For transgenic lines, describe the transformation method, the number of independent lines analyzed and the generation upon which experiments were performed. For gene-edited lines, describe the editor used, the endogenous sequence targeted for editing, the targeting guide RNA sequence (if applicable) and how the editor was applied.*
Authentication	*Describe any authentication procedures for each seed stock used or novel genotype generated. Describe any experiments used to assess the effect of a mutation and, where applicable, how potential secondary effects (e.g. second site T-DNA insertions, mosiacism, off-target gene editing) were examined.*

Flow Cytometry

Plots

Confirm that:

- [x] The axis labels state the marker and fluorochrome used (e.g. CD4-FITC).
- [x] The axis scales are clearly visible. Include numbers along axes only for bottom left plot of group (a 'group' is an analysis of identical markers).
- [x] All plots are contour plots with outliers or pseudocolor plots.
- [x] A numerical value for number of cells or percentage (with statistics) is provided.

Methodology

Sample preparation	Single-cell suspensions were derived from fresh mouse tumor tissues. Briefly, tumor tissues were cut into small pieces and physically passed through 100 μm strainers. Immune cells were enriched by density gradient centrifugation with Ficoll (StemCell, 07851). Cells were collected, washed and then stained with fluorescently conjugated antibodies. For cytokine detection, cell suspensions were incubated in culture medium containing PMA (5 ng/ml), ionomycin (500 ng/ml), Brefeldin A (1:1,000) and Monensin (1:1,000) at 37°C for 4 hours. These cells were subjected to cell surface and intracellular staining.
Instrument	Data collection was performed on a BDFortessa equipped with four lasers (BD Bioscience).
Software	All data were analyzed with FACS DIVA software v. 8.0 (BD Biosciences) or Flow Jo.
Cell population abundance	When cells were sorted or enriched, the purity was confirmed by flow cytometry and in each case the purity was above 90% .
Gating strategy	The cells were gated on FSC-A/SSC-A basis on the location known to contain lymphocytes and myeloid cells. Doublets were excluded based on FSC-A/FSC-H gating. Endogenous T cells were gated on CD45+CD3+CD8+ and analyzed for phenotype and cytokine production.

- [x] Tick this box to confirm that a figure exemplifying the gating strategy is provided in the Supplementary Information.
